# Supplementary figures and images for: Burkholderia pseudomallei type III secreted protein BipC: role in actin modulation and translocation activities required for the bacterial intracellular lifecycle
Source: PeerJ. 2016 Dec 21;4:e2532. doi: 10.7717/peerj.2532 (PMC5180589; doi:10.7717/peerj.2532)

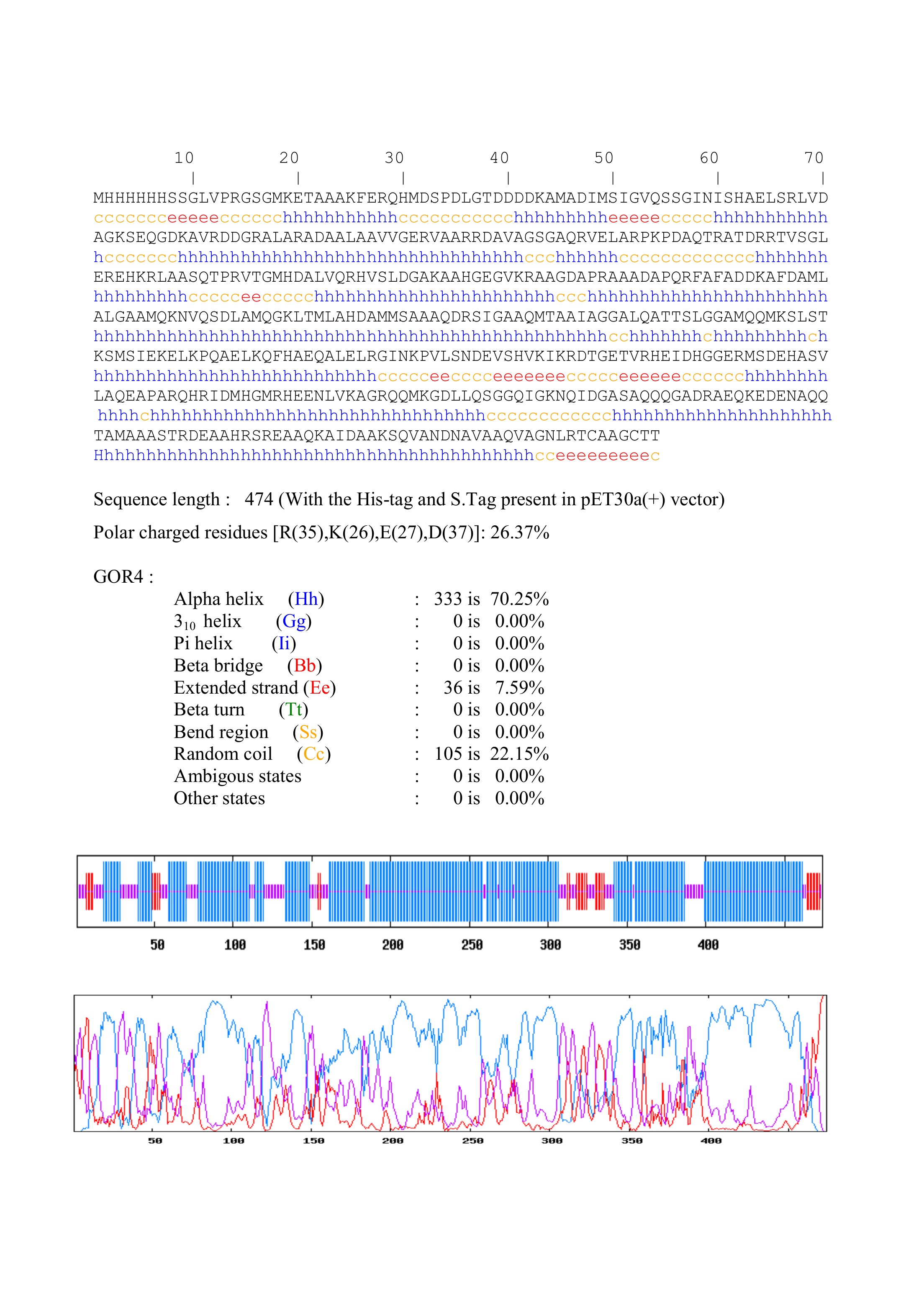

Supplement: Figure S1 — BipC displayed about 70.25% of alpha helix and 22.15% of random coil conformation. [file peerj-04-2532-s001.png]

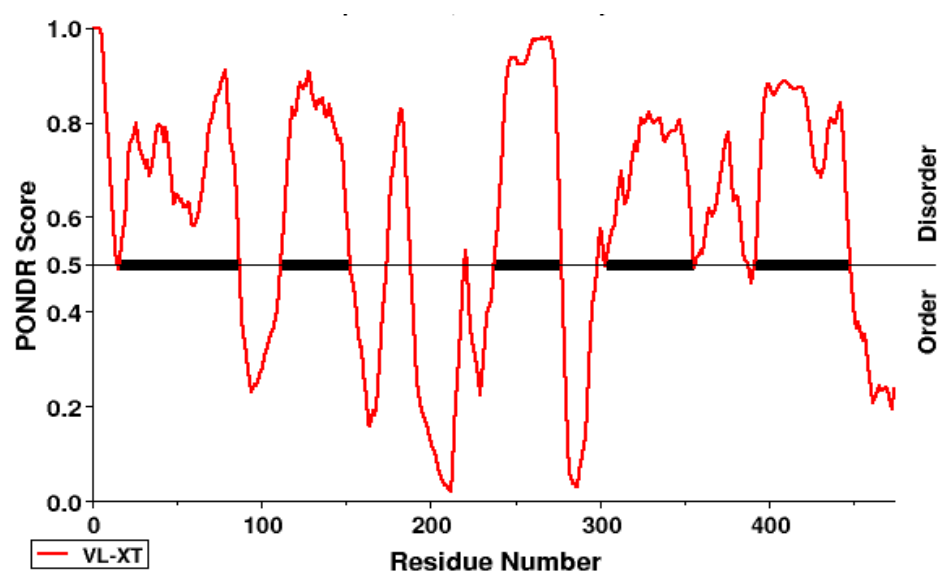

Supplement: Figure S2 — The x-axis represents residue number and the y-axis represents PONDR score. [file peerj-04-2532-s002.pdf]

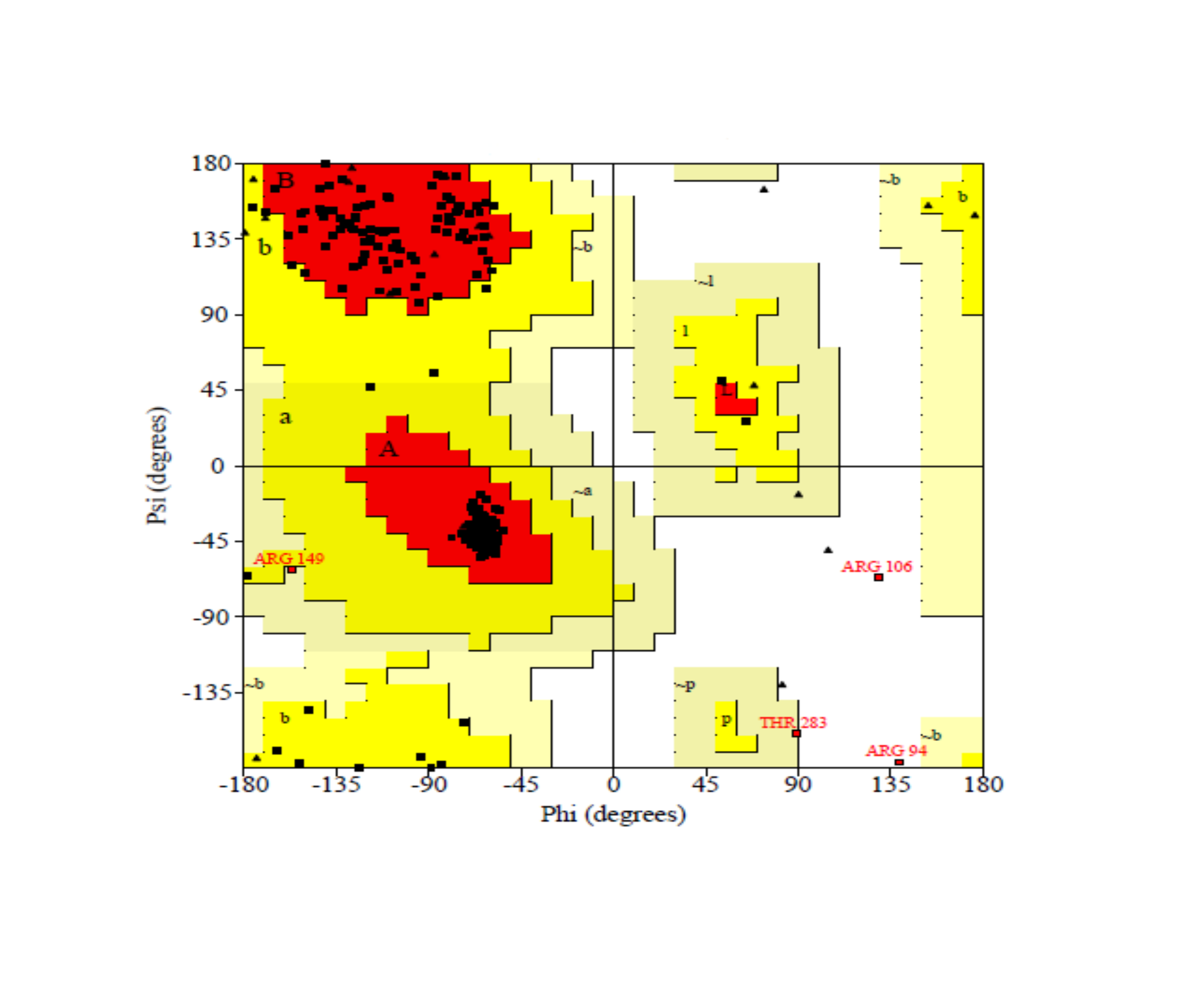

Supplement: Figure S3 — Red region represents the most favoured region (93.9%), yellow = allowed region (5.1%), light yellow = generously allowed region (0.5%), white = disallowed region (0.5%). [file peerj-04-2532-s003.png]
