# Supplementary material for: Burkholderia pseudomallei type III secreted protein BipC: role in actin modulation and translocation activities required for the bacterial intracellular lifecycle
Source: PeerJ. 2016 Dec 21;4:e2532. doi: 10.7717/peerj.2532 (PMC5180589; doi:10.7717/peerj.2532)
Supplement: Table S1 [file peerj-04-2532-s004.docx]

**Table S1. Strains and plasmids used in this study.**

| **Strains or plasmids** | **Relevant characteristic(s)** | **Source of reference** |
| --- | --- | --- |
| **Strains** |  |  |
| ***B. pseudomallei*** |  |  |
| K96243 | Wild type strain | (1) |
| ***E. coli*** |  |  |
| Top10 | Sp^r^, F- mcrA Δ(mrr-hsdRMS-mcrBC) φ80lacZ, ΔM15 ΔlacX74 nupG recA1 araD139 Δ(ara-leu)7697 galE15 galK16 rpsL endA1 λ^-^ | Invitrogen |
| BL21 (DE3) | fhuA2 [lon] ompT gal (λ DE3) [dcm] ∆hsdS, λ DE3 = λ sBamHIo ∆EcoRI-B int::(lacI::PlacUV5::T7 gene1) i21 ∆nin5 | Novagen |
| **Plasmids** |  |  |
| pCR^®^2.1-TOPO^®^ | Cloning vector, 3931bp, Ap^r^, Km^r^ | Invitrogen |
| pET-30a(+) | Expression vector, 5422bp, Km^r^ | Novagen |
| pC*_bipC_* | pCR^®^2.1-TOPO^®^ containing 1260 bp *bipC* fragment | This study |
| pET30a-*bipC* | pET30a (+) containing 1260 bp *bipC* fragment | This study |

Abbreviations: Ap, ampicillin; Km, kanamycin; Sp, spectinomycin; superscript r (^r^), resistant.

**Supplementary reference**

1. Holden MT, Titball RW, Peacock SJ, Cerdeno-Tarraga AM, Atkins T, Crossman LC, …, Parkhill J. 2004. Genomic plasticity of the causative agent of melioidosis, *Burkholderia pseudomallei*. *Proceedings of the National Academy of Sciences USA*. **101:** 14240-14245.
