# Supplementary material for: Burkholderia pseudomallei type III secreted protein BipC: role in actin modulation and translocation activities required for the bacterial intracellular lifecycle
Source: PeerJ. 2016 Dec 21;4:e2532. doi: 10.7717/peerj.2532 (PMC5180589; doi:10.7717/peerj.2532)
Supplement: Data S1 [file peerj-04-2532-s005.docx]

**Table Dataset 1:** Summary of pDomThreader z-score results in threading method.

| **Profile hit (PDB ID)** | **z-score** | **Confidence levels** |
| --- | --- | --- |
| 1wp1B01 | 5.02 | HIGH |
| 1yc9A01 | 3.54 | MEDIUM |
| 3g67A00 | 3.03 | MEDIUM |
| 1eq1A00 | 2.42 | MEDIUM |
| 1d66B02 | 2.04 | LOW |
| 1d7mA00 | 2.00 | LOW |
| 2v4hB00 | 1.89 | LOW |
| 1vykA00 | 1.89 | LOW |
| 1vsgA02 | 1.78 | LOW |
